# Tracking aluminium impurities in single crystals of the heavy-fermion superconductor UBe_13_

**DOI:** 10.1038/s41598-018-28991-w

**Published:** 2018-07-13

**Authors:** Alfred Amon, Iryna Zelenina, Paul Simon, Matej Bobnar, Marcel Naumann, Eteri Svanidze, Frank Arnold, Horst Borrmann, Ulrich Burkhardt, Walter Schnelle, Elena Hassinger, Andreas Leithe-Jasper, Yuri Grin

**Affiliations:** 1 Max-Planck-Institut für Chemische Physik fester Stoffe, Dresden, 01187 Germany; 2 Technische Universität München, Physik Department, Garching, 85748 Germany

## Abstract

The influence of Al incorporation on the heavy fermion superconductor UBe_13_ was investigated to explain the sample dependence of physical properties. Clear evidence for incorporated Al in flux-grown UBe_13_ single crystals is presented by results from X-ray diffraction, nuclear magnetic resonance and X-ray spectroscopy. The increase of the lattice parameter and the concomitant change of the superconducting properties are caused by substitution of Be in the compound by 1–2 at.% Al. The minute amounts of Al in the structure were located by atomic resolution transmission electron microscopy. Specific heat measurements reveal the strong influence of incorporated Al on the physical properties of UBe_13_. Upon long-term annealing, Al incorporated in single crystals can leave the structure, restoring properties of Al-free polycrystalline UBe_13_.

## Introduction

**Figure 1:**
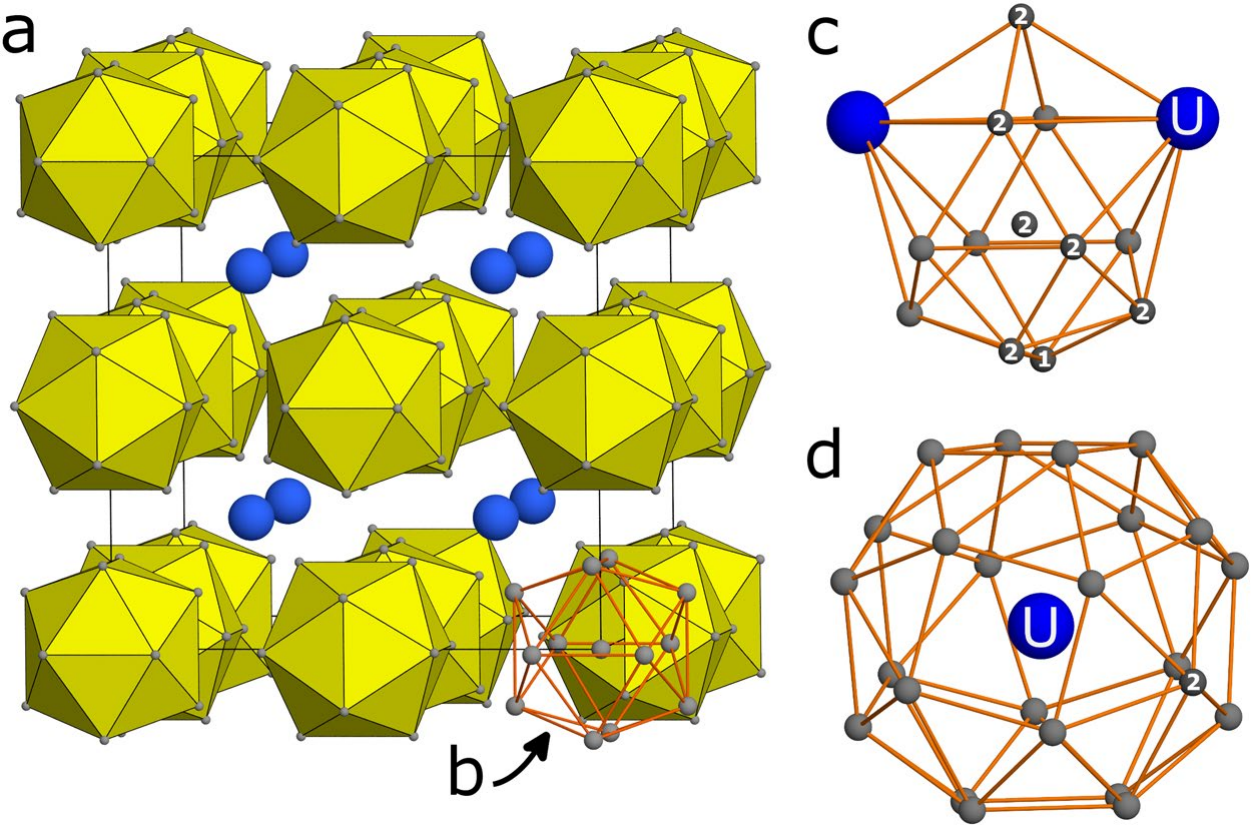
Crystal structure of UBe_13_, (**a**) CsCl-like arrangement of Be-centered Be_12_ icosahedra around U atoms (blue). Unit cell indicated by black lines. Coordination polyhedra of Be1 (**b**), Be2 (**c**) and U atoms (**d**).

Unconventional superconductivity usually appears near a suppressed magnetic phase, as is the case for many families of compounds - iron pnictides^1^, electron doped-cuprates^2^, organic superconductors^3^, and heavy fermion materials^4^. In these systems, the Cooper pairing mechanism is believed to be related to fluctuations of the magnetic order parameter^5^. UBe_13_ is a heavy fermion superconductor that stands out from this scheme, as no magnetic phase has yet been identified^6^ and hence the origin of the non-Fermi liquid behaviour and unconventional superconductivity remains a mystery. UBe_13_ crystallizes in the cubic NaZn_13_ structure type (space group $Fm\bar{3}c$) with the reported lattice parameter *a* = 10.254 Å (Fig. 1)^7,8^. Uranium at 8*a* site (1/4, 1/4, 1/4) is coordinated by 24 Be2 atoms at 96*i* site (0, 0.1763(1), 0.1150(1)). Be1 at 8*b* (0, 0, 0) is at the centre of an icosahedron formed by Be2 atoms^9^. The established route to large single crystals of UBe_13_ is the growth in aluminium flux, while polycrystalline UBe_13_ is accessible via arc melting of the elements^10^.

Extensive specific heat measurements have been performed for UBe_13_ on both single-^10–19^ and polycrystalline samples^20–33^, for pure as well as doped material^34–47^. Single crystal experiments have revealed an extremely complex and often contradictory behaviour of UBe_13_ in both the normal and superconducting state, evidenced by different shapes of the *H*_*c*2_(*T*) dependence, and small anomalies at different places in the *H* - *T* phase diagram^12,13,21,48^. Moreover, drastic variations in the lattice parameter *a*, the critical temperature *T*_*c*_, the normal state electronic specific heat coefficient *γ*, and the size of the specific heat anomaly Δ*C*_*p*_/*γT*_*c*_ have been observed for single and polycrystalline materials (Fig. S1)^49–51^. The lattice parameter *a* is reported to range from *a* = 10.254 Å to 10.2656 Å and the critical temperature from *T*_*c*_ = 0.35 K to 0.94 K^8,49,52^. Furthermore, *a* tends to be larger and *T*_*c*_ tends to be lower in single crystals, compared to polycrystalline samples. While this has been recognized previously, the possibility of Al incorporation from flux was excluded^49^. Spectroscopic measurements on single crystals also gave no indication of incorporated Al in UBe_13_^50,53^.

However, investigations of *H* - *T* phase diagrams as well as pressure studies found systematic differences in the physical properties between single crystals and polycrystalline samples^14,16,54^. Furthermore, a clear correlation between the lattice parameter *a* and the Al content *x* in polycrystalline UBe_13−*x*_Al_*x*_ was established^52,55^. Recent specific heat measurements revealed a decrease of *T*_*c*_ upon Al addition in polycrystalline samples^56^. Nonetheless, no conclusive answer about the possibility and effect of Al incorporation in UBe_13_ has yet been provided^50,51^. To address these contradictory experimental observations, it was suggested that two variants of UBe_13_ exist: “H-type” (high *T*_*c*_, 0.85 K < *T*_*c*_ < 0.95 K) and “L-type” (low *T*_*c*_, *T*_*c*_ < 0.75 K). The “L-type” behaviour was found to occur only in single crystals, suggesting Be content variation or possible Al incorporation as the underlying cause, while the “H-type” behaviour was found for both single crystals and polycrystalline materials^13^.

The aim of this investigation is to clarify the following issues: (i) is Al incorporated into the crystal structure of UBe_13_, and if so, what is the solubility limit of Al in UBe_13−*x*_Al_*x*_; (ii) where is Al located within the crystal structure; (iii) what is the influence of Al substitution on the physical properties of UBe_13_ and what is the effect of annealing on incorporated Al. These questions are addressed in the current study by means of powder X-ray diffraction (PXRD), single crystal X-ray diffraction experiments (SXRD), wavelength dispersive X-ray spectroscopy (WDX), ^27^Al nuclear magnetic resonance (NMR) spectroscopy, transmission electron microscopy (TEM), as well as specific heat measurements.

## Results

### Solubility of Al in UBe_13−*x*_Al_*x*_

**Figure 2:**
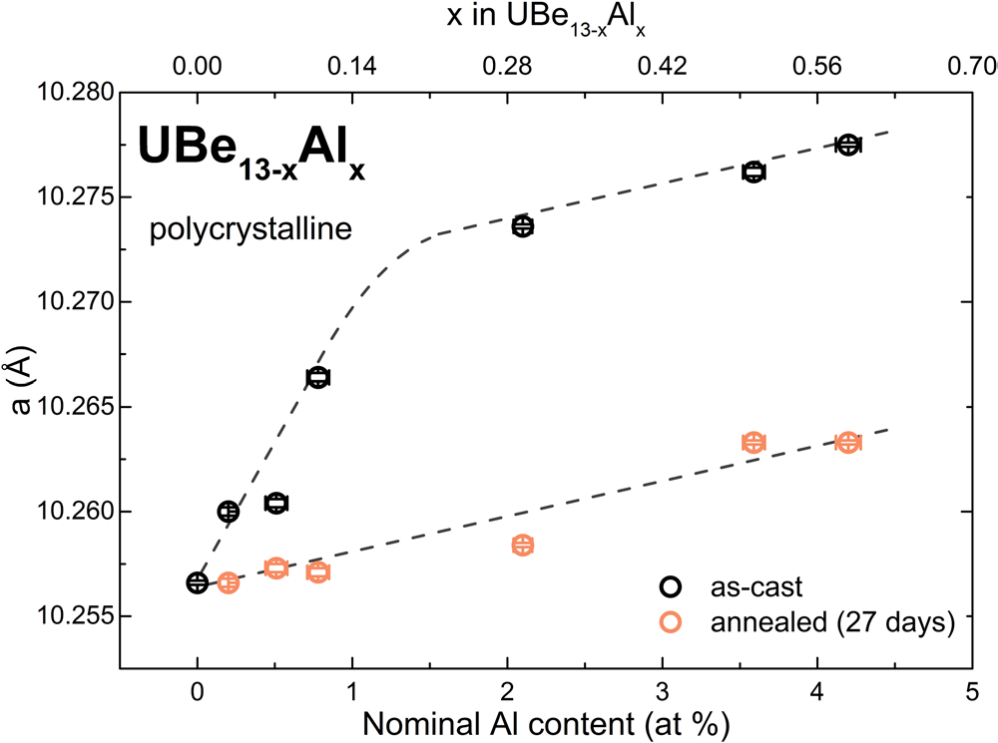
Lattice parameter *a* as a function of the nominal Al content in as-cast (black) and annealed (orange) polycrystalline UBe_13−*x*_Al_*x*_. The dashed lines are guides to the eye.

A substitution series of polycrystalline UBe_13−*x*_Al_*x*_ (0 ≤ *x* ≤ 0.60) samples was prepared in order to investigate the possible solubility of Al in UBe_13_. On addition of Al, the lattice parameter *a* of UBe_13−*x*_Al_*x*_ increases linearly from 10.2566(1) Å (*x* = 0) up to 10.2736(1) Å (*x* = 0.31), where the increase starts to saturate (Fig. 2). For *x* > 0.07 and *x* > 0.31, small amounts of UAl_2_ and UAl_3_ are present in the powder diffraction data, respectively. Annealing of the UBe_13−*x*_Al_*x*_ samples significantly decreases the lattice parameter for all compositions (Fig. 2, measured lattice parameters listed in Table S1). The relative amounts of UAl_2_ and UAl_3_, observed in PXRD, increase upon annealing. The increase of the lattice parameter with Al content indicates the incorporation of Al into UBe_13_, forming the substitutional solid UBe_13−*x*_Al_*x*_. The change in the slope of the curve *a* vs. Al content, together with the appearance of UAl_2_ and UAl_3_ reveals the non-equilibrium state of the material. At temperatures around the melting point, Al is incorporated into UBe_13−*x*_Al_*x*_, leading to a lattice expansion. As the temperature is lowered, the solubility decreases and, during annealing Al atoms diffuse and leave the crystal. It has been argued that the incorporated Al may be distributed statistically on the Be1 and Be2 sites in the as-cast state of the samples and then orders on the Be1 sites upon annealing, instead of leaving the structure^55^. Results of specific heat and WDX measurements after annealing speak against an ordering of Al in the structure (see below). The lattice parameter of flux-grown single crystals of UBe_13−*x*_Al_*x*_ is significantly larger compared to Al-free polycrystalline samples (Table S2), indicating Al incorporation from flux.

**Figure 3:**
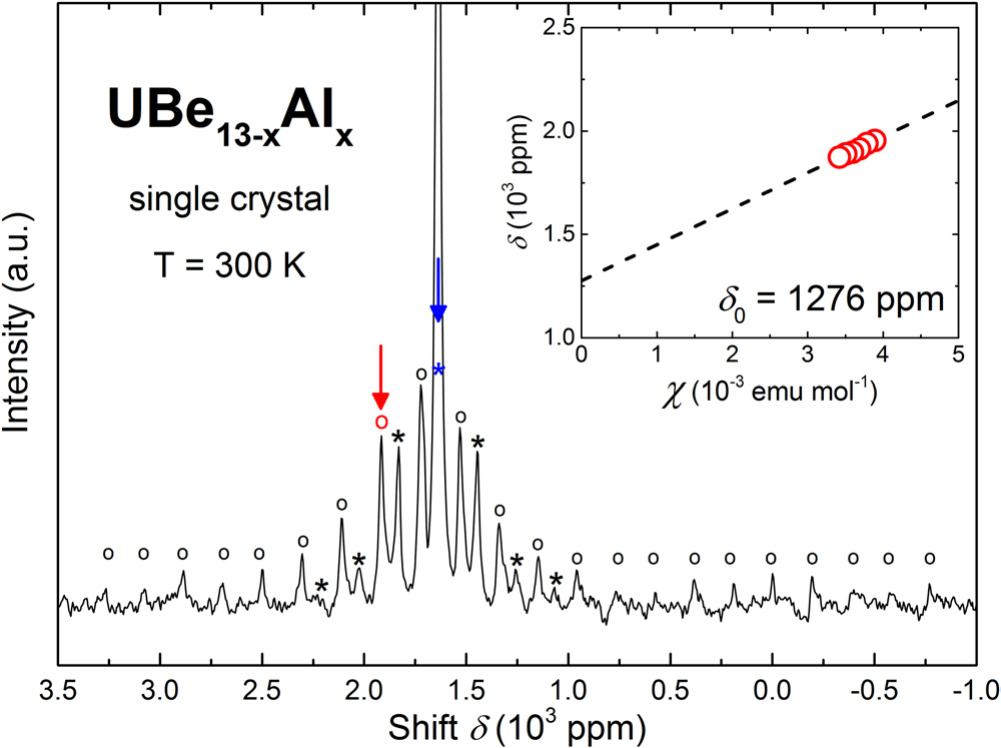
^27^Al NMR (MAS) spectrum of a powdered single crystal of UBe_13−*x*_Al_*x*_ in as-grown condition. Central lines for Al incorporated in UBe_13−*x*_Al_*x*_ (central line *δ* = 1915 ppm, circles) and Al flux residue (*δ* = 1635 ppm, asterisks) are indicated by red and blue arrows, respectively. Inset: Temperature dependence of the Al-signal shift in UBe_13−*x*_Al_*x*_ (red circles), scaled as *χ*(*T*) = 2.38*μ*_*B*_/(*T* + 85)^20^, along with the linear extrapolation of the data (dashed, black line).

**Figure 4:**
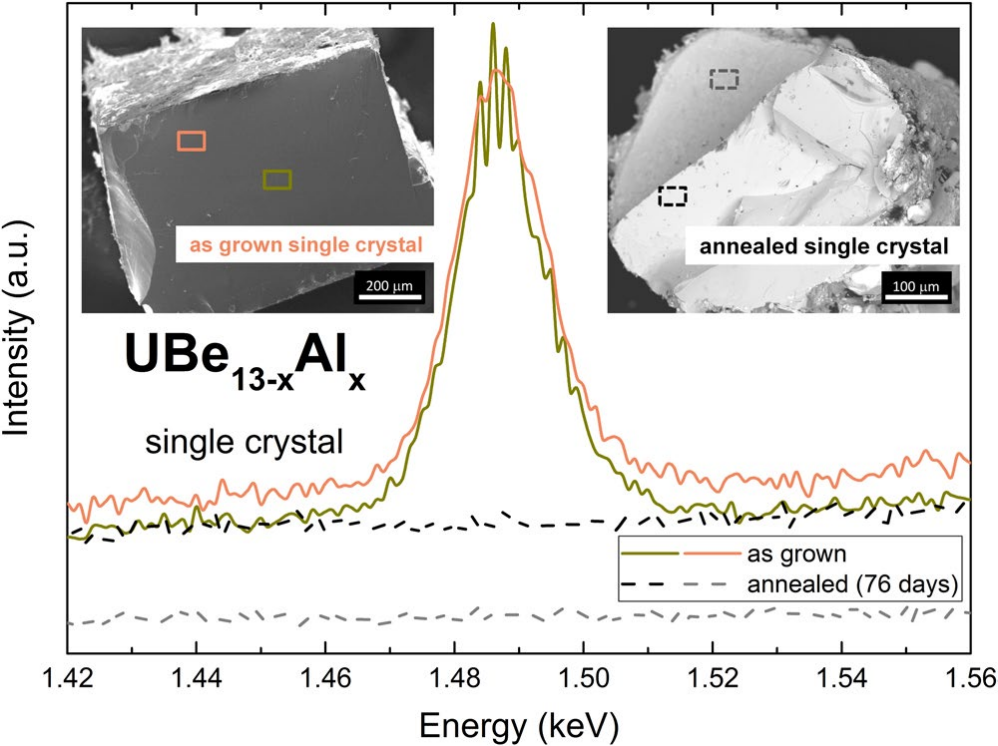
WDX spectra of the Al-K*α* line (*E* = 1.49 keV), recorded on surfaces of as-grown (solid lines) and annealed (dashed lines) single crystals of UBe_13−*x*_Al_*x*_. After long-term annealing no Al is detectable. Insets: SEM images of UBe_13−*x*_Al_*x*_ crystals, with corresponding measurement spots marked by rectangles.

Magic angle spinning (MAS) ^27^Al nuclear magnetic resonance (NMR) spectroscopy on powdered single crystals of UBe_13−*x*_Al_*x*_ reveals two sharp, strongly overlapping signals (Fig. 3). The signal with a Knight shift *δ* = 1915 ppm (*T* = 300 K) cannot be attributed to any of the known phases in the U–Be–Al system (cf. Fig. S3), and originates from Al incorporated in UBe_13−*x*_Al_*x*_. It overlaps with a strong signal from Al metal flux residue (*δ* = 1635 ppm). A clear temperature dependence of the isotropic Knight shift was found for the signal with *δ* = 1915 ppm, consistent with paramagnetism in UBe_13_ (inset of Fig. 3). Pulse length optimization for this signal reveals that about 2 to 3 times shorter pulses are needed for maximum intensity compared to the signal of Al metal, due to the spread out of the satellite transitions. This indicates non-zero quadrupolar coupling, which is a sign of non-cubic site symmetry for Al in UBe_13−*x*_Al_*x*_, excluding substitution on the Be1 site at the icosahedron center^57,58^. This is in contrast to B-substituted UBe_13−*y*_B_*y*_, where the smaller B atoms were reported to substitute exclusively at the cubic Be1 site^59^. Wavelength dispersive X-ray spectra (WDX), recorded on surfaces of freshly cleaved UBe_13−*x*_Al_*x*_ single crystals, show the Al-K*α* line (*E* = 1.49 keV), confirming the incorporation of Al in the crystal structure (Figs 4 and S4). After long term annealing, the Al signal is below the limit of detection, corroborating that Al leaves the crystal. Scanning electron microscopy of the cross section of the annealed crystal (inset to Fig. 4) reveals that a thin layer formed on the surface of the crystal. Unfortunately, the small dimensions have prevented a detailed investigation of the composition up to now.

### Locating Al in the crystal structure of UBe_13−*x*_Al_*x*_

**Figure 5:**
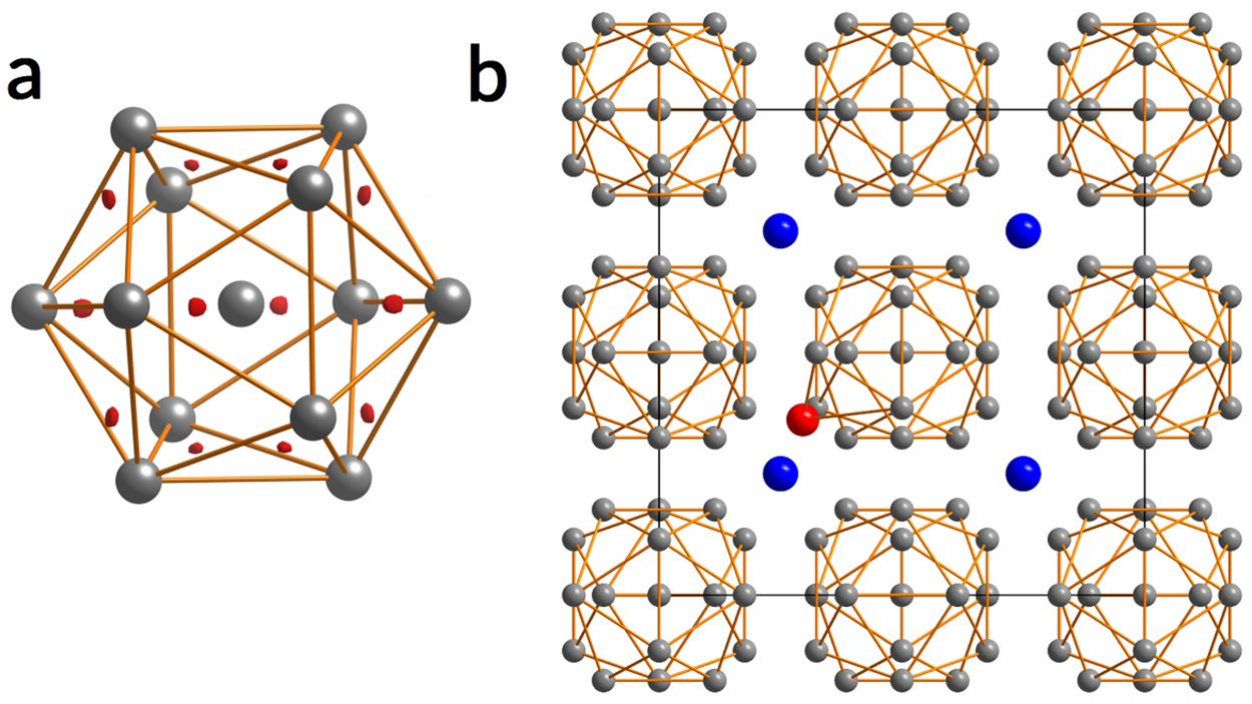
(**a**) Be@Be_12_ structural unit with residual electron density maxima, shown by a red isosurface at 1.7 e Å^−3^, in the Al-free structure model from single crystal X-ray diffraction. (**b**) Unit cell projection along $[1\,0\,0]$ of UBe_13−*x*_Al_*x*_ with one possible Al site realized. U, Be and Al atoms drawn as blue, grey and red spheres, respectively.

Refinement of high-quality single crystal diffraction data for as-grown UBe_13−*x*_Al_*x*_ agrees well with the published structure model^9^. However, careful investigation of the residual electron density reveals additional peaks. The observed maxima are located at the Be-icosahedron face, close to the Be2 position (Fig. 5a). This residual density was accounted for by Al replacing 1.8% of the Be2 in the icosahedron, improving the figure of merit *R*_*F*_ from 1.17% to 1.01% (Table 1). Thereby, the Al and Be position slightly differ due to the size difference between Be and Al (*r*_*Be*_ = 1.12 Å, *r*_*Al*_ = 1.43 Å^60^). The occupation of the Al position in this crystal yields 1 to 2 Al atoms per unit cell, on average (Fig. 5b). This scenario explains the reported disorder on the Be2 site, which manifests itself in strongly increased thermal parameters, observed by neutron powder diffraction of flux-grown UBe_13−*x*_Al_*x*_ single crystals^9,61^.

**Table 1:** Crystallographic details, atomic coordinates, displacement parameters and occupation factors of UBe_13−*x*_Al_*x*_ (*x* = 0.21) from single crystal diffraction.

Refined composition	UBe_12.79(5)_Al_0.21(5)_
Crystal system, space group	Cubic, $Fm\bar{3}c$
Formula units per cell	8
2*θ*_*max*_	85.8°
N(hkl)_*measured*_	5815
N(hkl)_*unique*_	190
N(hkl)_*observed*_ (*F*_*hkl*_ > (4*σ*(*F*)))	190
*R*_*int*_/*R*_*σ*_	0.029/0.008
*R*_*F*_/*wR*_*F*2_	0.010/0.014
Refined parameters	9
Weighting scheme	*w*_*i*_ = ${[\mathrm{ln}({F}_{obs,i}^{4})]}^{-1}$
Residual electron density maxima	+0.39/−0.30 e Å^−3^
Atom, site	*x*/*a*, *y*/*b*, *z*/*c*, *B*_*iso*_/Å^2^, occupancy
U1, 8*a*	1/4, 1/4, 1/4, 0.408(3), 1
Be1, 8*b*	0, 0, 0, 0.49(11), 1
Be2, 96*i*	0.1759(3), 0.1147(3), 0, 0.4551*, 0.982(4)
Al1, 192*j*	0.204(5), 0.139(5), 0.519(5), 0.4551*, 1/2 × (1 − 0.982)

*B_*iso*_(Be2) = *B*_*iso*_(Al1).

**Figure 6:**
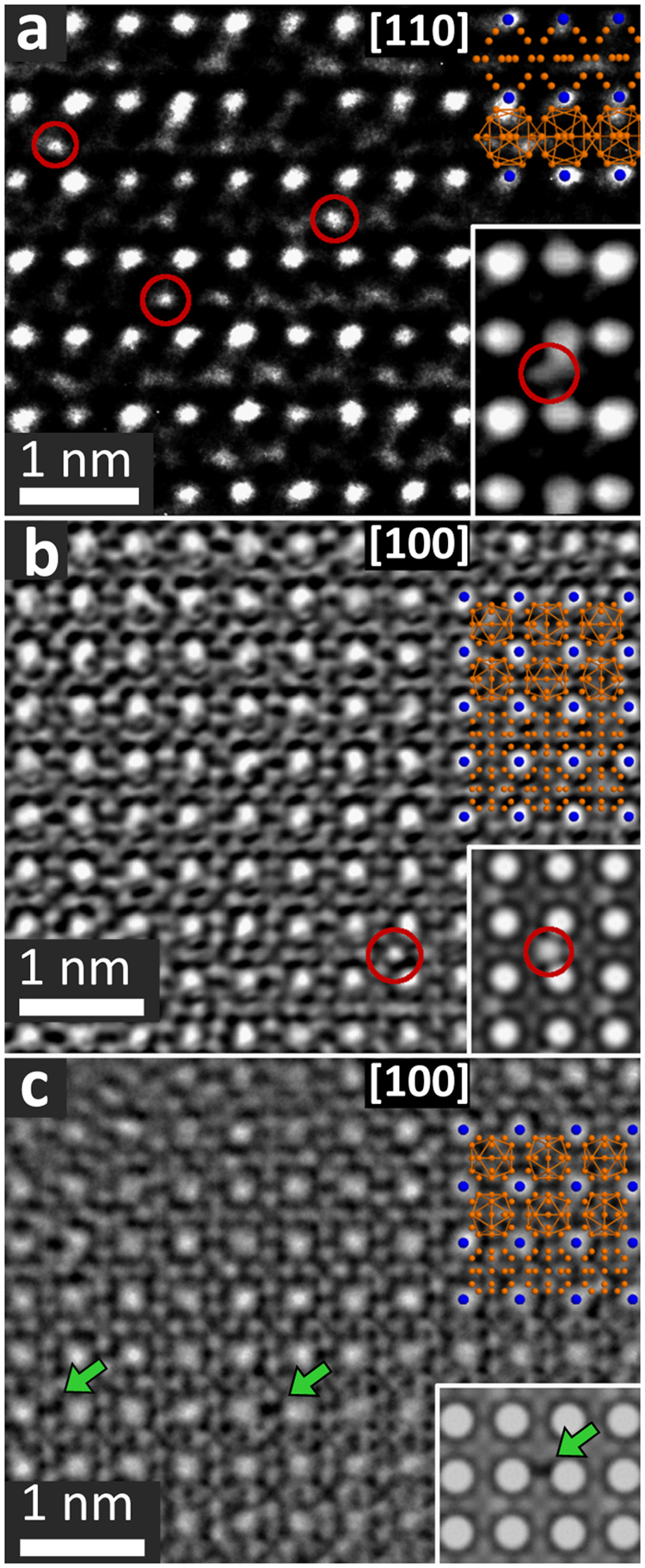
HRTEM of a UBe_13−*x*_Al_*x*_ single crystal in (**a**) as-grown condition: $[1\,1\,0]$ zone, (**b**) after 38 d annealing: $[1\,0\,0]$ zone, (**c**) after 76 d annealing: $[1\,0\,0]$ zone. Al atoms are exemplarily highlighted by red circles, Be vacancies by green arrows. The idealized crystal structure is overlaid at the right hand side of the panels. Insets in (**a**,**b**) show simulation of Al substituting Be2. Inset in (**c**) shows simulation of a Be2 vacancy.

High-resolution transmission electron microscopy (HRTEM) was performed on single crystals of UBe_13−*x*_Al_*x*_ after crystal growth and after two annealing steps to localize the Al incorporations. HRTEM images of as-grown UBe_13−*x*_Al_*x*_ show a rather homogeneous crystal lattice (Fig. 6a). Detailed inspection reveals strong contrast variations between the U atoms due to Al substitution on the Be lattice. The Al substitution can be clearly assigned to the Be2 site, as confirmed by TEM image simulations. Residual Al incorporations in a uniform crystal lattice are still detectable after 38 d of annealing (Fig. 6b). After 76 d of annealing, the Be substructure is dominated by strong contrast variations, which can be explained by accumulation of Be vacancies (Fig. 6c). The vacancies arise from the expulsion of Al and Be from the crystal. Furthermore, an apparent degradation on the nano-scale was observed for several of the investigated crystallites in this sample (Fig. S5), in line with the sample degradation effects observed in specific heat data (see below).

### Superconductivity and annealing effects of UBe_13−*x*_Al_*x*_

**Figure 7:**
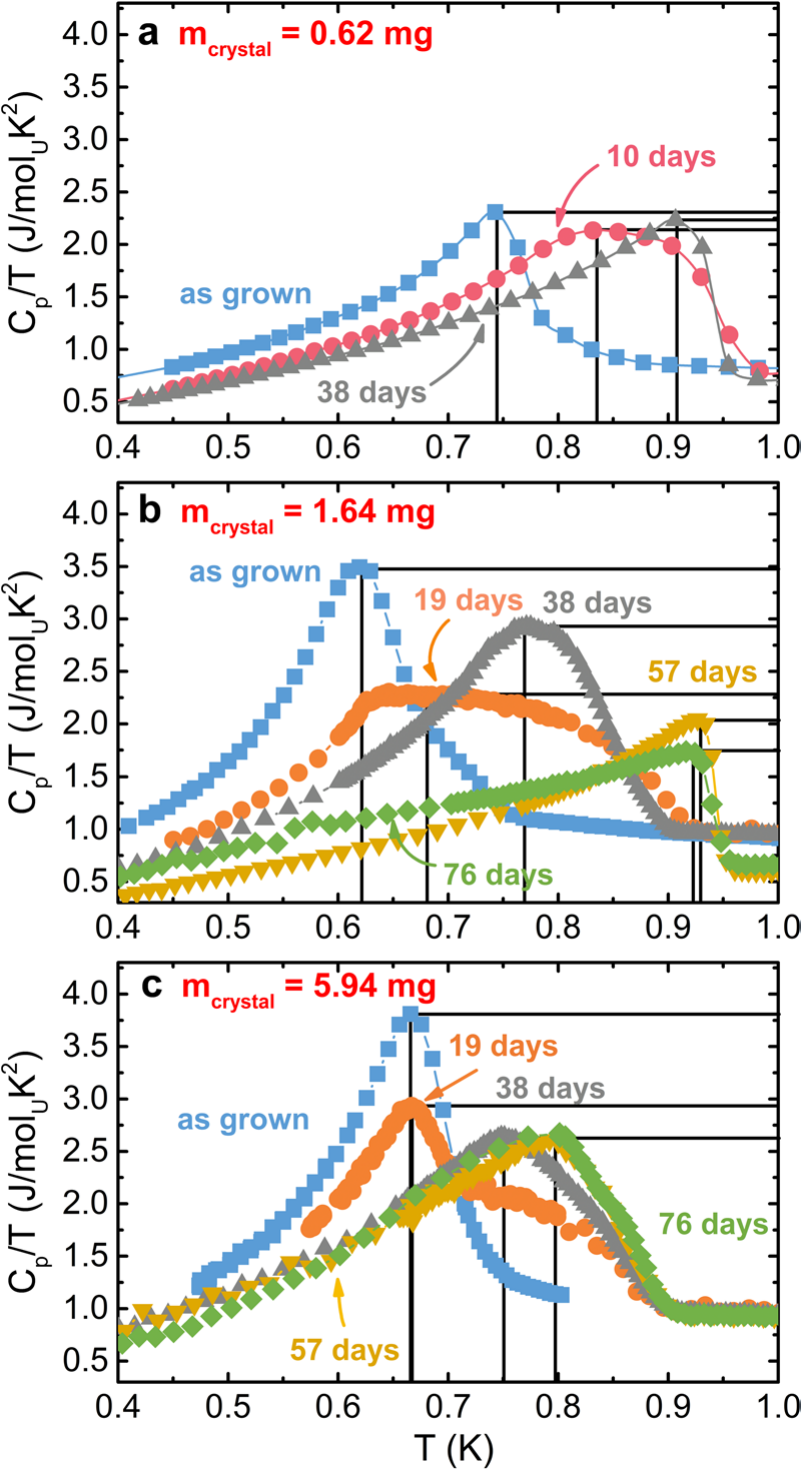
*C*_*p*_/*T* as a function of *T* for three UBe_13−*x*_Al_*x*_ single crystals of different size at different stages of annealing. The maximum in *C*_*p*_/*T* is marked by black lines.

The effects of annealing on the low-temperature specific heat of UBe_13−*x*_Al_*x*_ were investigated on three single crystals of different size (0.62 mg, 1.64 mg, 5.94 mg; cf. Methods Section). In as-grown state, the small, medium and large crystal show *T*_*c*_ = 0.78 K, *T*_*c*_ = 0.66 K and *T*_*c*_ = 0.70 K, respectively (Fig. 7). Upon annealing, the onset of the superconducting transition shifts toward *T* = 0.9 K for all three crystals, accompanied by a strong broadening of the transition and a decrease of the jump Δ*C*_*p*_/*γT*_*c*_. For the small crystal, the width of the superconducting transition decreases again upon further annealing and *T*_*c*_ increases to the high value of 0.94 K, in line with reports that the annealing of UBe_13−*x*_Al_*x*_ single crystals increases *T*_*c*_^55,56^. For the medium and large crystals several stages are distinguishable after 19 d, 38 d and 57 d. The broadened signal appears to reflect at least two overlapping transitions with a range of transition temperatures.

Upon annealing, the entropy associated with the low *T*_*c*_ transitions is reduced and transferred to the high *T*_*c*_ transitions. Integration of the peak in *C*_*p*_/*T* up to 1 K reveals a nearly constant area up to 38 d for the small and medium and up to 76 d for the large crystal. The small and medium-sized crystals reach a final state after 38 d and 57 d of annealing, respectively, characterized by a single transition with a sharp onset. The ratio Δ*C*_*p*_/*γT*_*c*_ and also the Sommerfeld coefficient *γ* of the normal conducting state are reduced compared to the as-grown state (cf. Fig. S6). The large crystal shows a slower change than the small and medium crystals and has not reached a comparable final state even after 76 d of annealing. The crystal-size dependence of the annealing effect corroborates the diffusion-based origin of the observed behaviour. The increased *T*_*c*_ and reduced specific heat anomaly Δ*C*_*p*_/*γT*_*c*_, observed for the final state, are concomitant with a decrease of the lattice parameter (from *a* = 10.2629(1) Å to *a* = 10.2579(2) Å) and Al depletion as evidenced by WDX spectroscopy on the medium-sized crystal (Fig. 4).

**Figure 8:**
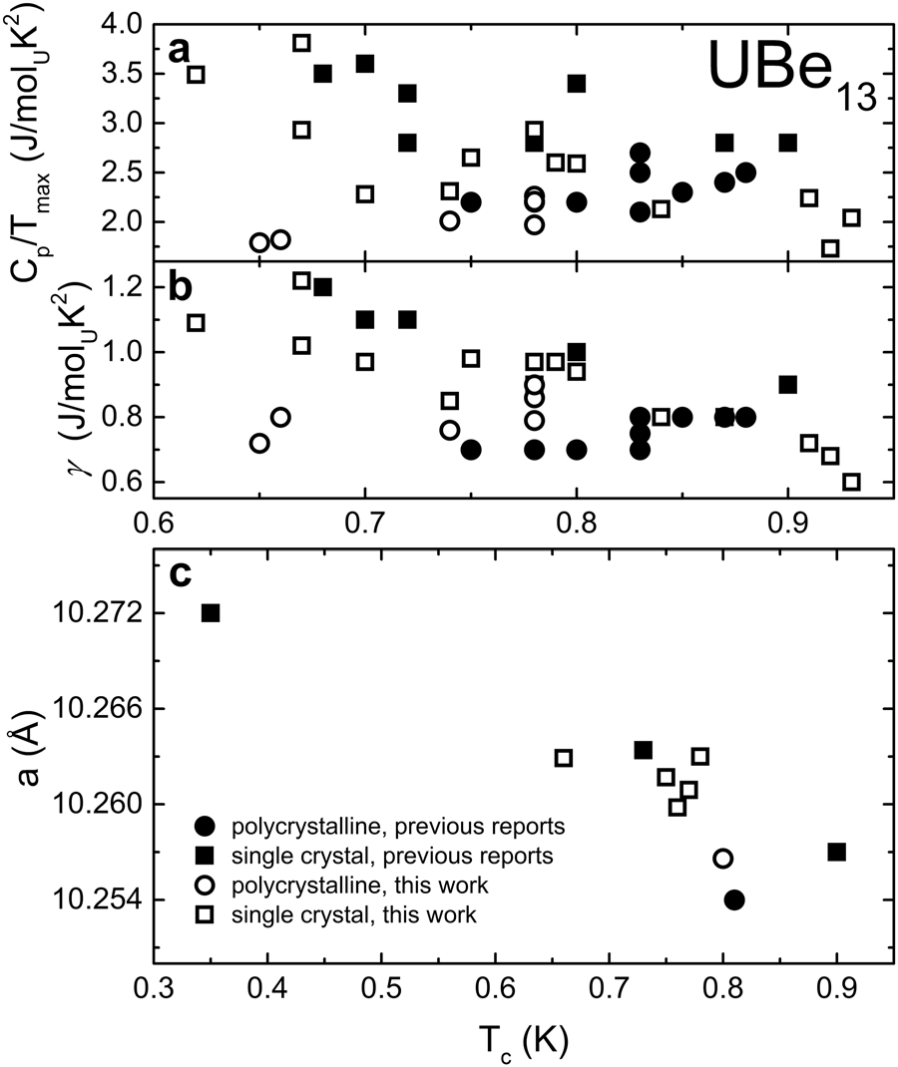
The peak maxima in *C*_*p*_/*T* at the superconducting transition (**a**), the electronic specific heat coefficient *γ* (**b**) and the lattice parameter *a* (**c**) plotted against the critical temperature *T*_*c*_ for single crystals (squares) and polycrystalline samples (circles) of UBe_13_. Data from this work and literature reports^10–34^ (cf. Table S3) are drawn as empty and full symbols, respectively.

Considering the incorporated Al as an electron donor in UBe_13−*x*_Al_*x*_, the enhanced electronic specific heat in the as-grown state could be a result of an increased charge carrier concentration, in line with theoretical and experimental band structure studies^62–64^. In this respect, the reduced size of Δ*C*_*p*_/*γT*_*c*_ ≈ 2.0–2.3 in the final state is comparable to reports of Al-free polycrystalline UBe_13_^34,46^. The increased jump Δ*C*_*p*_/*γT*_*c*_, observed in several UBe_13−*x*_Al_*x*_ single crystals, is reminiscent of substitution experiments with the isoelectronic element B in UBe_13−*y*_B_*y*_, where the addition of B led to drastic changes in the low temperature behaviour of the electronic specific heat^36,37,46,55^. The observed changes of the critical temperature *T*_*c*_, size of *C*_*p*_/*T*_*max*_, Sommerfeld coefficient *γ* and lattice parameter *a* cover the range of reported values for various samples of UBe_13_ (Fig. 8). Therefore, the reported variations in the superconducting properties can be ascribed to the respective sample history, i.e. different annealing treatments and Al contents.

Annealing of the medium sized crystal to a total of 76 d did not increase *T*_*c*_ any further, but led to a decrease of the jump in specific heat, a decrease of the transition peak area and an even flatter decay towards lower temperatures. We take this as indication that prolonged annealing can lead, besides depletion of Al, to a partial disintegration of the crystal structure, accompanied by the accumulation of vacancies in the Be substructure. This is in agreement with the observed variations of the lattice parameter in Al-free, off-stoichiometric, polycrystalline samples, ranging from *a* = 10.2564(2) Å (UBe_11_) to *a* = 10.2592(1) Å (UBe_15_).

## Discussion

The dependence of the lattice parameter *a* on Al content for as-cast polycrystalline samples UBe_13−*x*_Al_*x*_ confirms the incorporation of Al in UBe_13_. Wavelength dispersive X-ray spectroscopy on inner surfaces of cleaved UBe_13−*x*_Al_*x*_ single crystals unequivocally shows the presence of aluminum in Al-flux grown crystals. The temperature dependence of the ^27^Al NMR signal (*δ*(300 K) = 1915 ppm) of a UBe_13−*x*_Al_*x*_ single crystal corroborates the signal origin from Al in the UBe_13_ structure, while the pulse-length dependence of the ^27^Al signal indicates non-cubic site symmetry for Al in UBe_13−*x*_Al_*x*_. Additional electron density peaks at the Be icosahedron face close to the Be2 position were observed in single crystal X-ray diffraction, revealing that Al replaces Be2 in the structure (1–2 Al atoms per unit cell). Transmission electron microscopy images show indeed Al atoms substituting on the Be2 position. The decrease of the lattice parameter upon annealing of the polycrystalline samples UBe_13−*x*_Al_*x*_ indicates a strong temperature dependence of the Al solubility. Al is incorporated and trapped during the solidification of UBe_13−*x*_Al_*x*_, but can diffuse and leave the structure upon long-term annealing, as reflected by the formation of binary U–Al compounds.

Annealing strongly influences the low temperature specific heat and superconducting transition of UBe_13−*x*_Al_*x*_ single crystals. Long-term annealing leads to an increase of *T*_*c*_ above 0.9 K, where the transition seems to shift in several stages. The diffusion of Al to the surface of the crystal during annealing creates volumes with varying Al content which is reflected in a spread of transition temperatures *T*_*c*_ within one crystal. Annealing can therefore change the behaviour of an “L-type” crystal to “H-type”, equivalent to a decrease in Al content. Long-term annealing leads to a uniform Al depletion, as evidenced by a sharp transition in specific heat and WDX spectra of the annealed crystal. The low-temperature specific heat of annealed single crystals behaves similar to Al-free polycrystalline samples. The annealing time needed to reach the highest *T*_*c*_ is proportional to the crystal size, confirming that Al diffuses and leaves the crystal rather than ordering locally on a specific site. However, prolonged annealing may lead to a degradation of the structure due to the relatively high Be vapour pressure. This is evidenced by the decrease of superconducting phase fraction in specific heat and the formation of vacancies in the Be lattice observed in HRTEM.

## Methods

The polycrystalline samples of UBe_13−*x*_Al_*x*_ (0 ≤ *x* ≤ 0.60), as well as the binary compounds UAl_2_ and UAl_3_ (NMR reference material) were synthesized by arc melting of the elemental metals, using U (sheet, Good Fellow, >99.95 wt.%), Be (sheet, Heraeus, >99.9 wt.%), and Al (droplets, Chempur, 99.999 wt.%). A small excess of Be was added to compensate for the material loss due to evaporation (typically 1.5–1.7 wt.%). Complete sample preparation was performed in Ar-filled glove boxes (MBraun, p(H_2_O/O_2_) < 0.1 ppm), dedicated to the handling of U- and Be-containing samples^65^. The polycrystalline samples were annealed in alumina crucibles under Ar atmosphere.

Large, mm-sized, cube-shaped single crystals of UBe_13−*x*_Al_*x*_ were grown using Al flux. Arc melted specimens of U with Be and Al (U:Be:Al atomic ratio of 1:18:35) were placed in a BeO crucible. The crucible was then sealed under Ar atmosphere in a tantalum tube and placed in a furnace (LORA, HTM Reetz). The samples were heated up and kept at 1500 °C for 72 h and then cooled at 3 °C h^−1^ to room temperature. The crystals were isolated by selective dissolution of the Al flux in an aqueous NaOH solution (2 mol L^−1^).

The effects of annealing were investigated on three single crystals of UBe_13−*x*_Al_*x*_. The smallest crystal (0.62 mg) was isolated from Al flux and the two larger pieces (1.64 mg and 5.94 mg) were cut from one large single crystal (Fig. S2). The crystals were repeatedly annealed at 900 °C for intervals of 10 d to 19 d and the low temperature specific heat was measured after each annealing step. The single crystals were annealed in BeO crucibles, with added polycrystalline powder of UBe_13_, covered with a Be lid and sealed in a tantalum tube to guarantee sufficient Be vapour pressure in order to avoid Be losses during the heat treatment.

X-ray powder diffraction was performed on a Huber G670 Image Plate Guinier Camera equipped with a Ge-monochromator (Cu-K*α*1 radiation, *λ* = 1.54056 Å). Single crystal diffraction data were recorded on a Rigaku AFC7 diffractometer equipped with a Saturn724+ CCD detector (Mo-K*α*) (Details in Table 1). Indexing of powder diffraction patterns, structure solution based on single crystal diffraction data and structure refinement were performed within the WINCSD program package^66^. Lattice parameters were determined from powder X-ray diffraction by a least-squares refinement of the peak positions using an internal standard (LaB_6_). The heat capacity *C*_*p*_ was measured by thermal-relaxation calorimetry on a Quantum Design PPMS, equipped with a ^3^He cooling system.

Magic angle spinning (MAS) ^27^Al nuclear magnetic resonance (NMR) experiments were performed on a Bruker Avance 500 spectrometer with a magnetic field of *B*_0_ = 11.74 T and a standard Bruker MAS probe for 2.5 mm ZrO_2_ rotors at a spinning frequency of 25 kHz. The samples were powdered and diluted with dry KCl in order to increase the accessible surface area and therefore the signal intensity, which is limited by the penetration depth of the oscillating magnetic field in metallic samples. The reference frequency for ^27^Al was 130.31572 MHz, corresponding to the signal of an Al(NO_3_)_3_ solution in D_2_O. The spectra were obtained from the echoes after two pulses (${90}_{x}^{^\circ }$ − *τ* − (90° or 180°)_*y*_ − *τ* − acquisition) and recovery times between 50 ms and 500 ms. The presented spectr°um in Fig. 3 is a sum of three spectra where the observation window was centered at the isotropic position of the signal and shifted for ±150 kHz in frequency. In this way, the rotational sidebands spreading from about −1000 ppm to about +3000 ppm could be observed. The isotropic line at *δ* = 1915 ppm was determined by varying the MAS spinning rate. The sample temperature was obtained from the measured temperature in the MAS probe and the estimated temperature increase due to the frictional heating of the spinning rotor. The temperature-independent Knight shift *δ*_0_, due to the conduction electrons, was evaluated by linear extrapolation of the isotropic shift *δ* vs. magnetic susceptibility to *χ* = 0.

Scanning electron microscopy was performed on a JEOL JSM-6610 scanning electron microscope (15 kV acceleration voltage) equipped with a secondary electron detector, an electron backscatter detector, a ThermoScientific UltraDry EDX detector and a ThermoScientific MagnaRay WDX spectrometer. Wavelength dispersive X-ray spectroscopy (WDX) was performed on several crystals from different synthesis batches as an independent method to prove the presence of Al in the crystals and to separate the Al line from the U-escape peak of the EDX-Si detector.

High-resolution TEM (HRTEM) analyses were performed on the JEM-ARM300F (Grand ARM, JEOL) with double correction. The spherical aberration of the condenser and the objective lens are corrected by dodecapole correctors in the beam and the image forming system. TEM resolution is 0.5–0.7 Å depending on resolution criterion applied. TEM images were recorded on a 4 k × 4 k pixel CCD array (Gatan US4000). Additional analysis was carried out on a FEI Tecnai F30-G2 with Super-Twin lens (FEI) with a field emission gun at an acceleration voltage of 300 kV. The point resolution amounted to 2.0 Å, and the information limit to about 1.2 Å. The microscope was equipped with a wide angle slow scan CCD camera (MultiScan, 2 k × 2 k pixels; Gatan Inc., Pleasanton, CA, USA). The datasets generated or analysed during the current study are available from the corresponding author on reasonable request.

## Electronic supplementary material

Supplementary Information
