# Supplementary material for: Tracking aluminium impurities in single crystals of the heavy-fermion superconductor UBe13
Source: Sci Rep. 2018 Jul 13;8:10654. doi: 10.1038/s41598-018-28991-w (PMC6045676; doi:10.1038/s41598-018-28991-w)
Supplement: Supplementary file 1 — Supplementary Information [file 41598_2018_28991_MOESM1_ESM.pdf]

Supporting Information for the article:
Tracking aluminum impurities in single crystals of the heavy-fermion superconductor UBe_{13}.

Alfred Amon[†*], Iryna Zelenina[†], Paul Simon[†], Matej Bobnar[†], Marcel Naumann[†], Eteri Svanidze[†], Frank Arnold[†], Horst Borrmann[†], Ulrich Burkhardt[†], Walter Schnelle[†], Elena Hassinger[†], Andreas Leithe-Jasper[†*], and Yuri Grin[†]

[†] Max-Planck-Institut für Chemische Physik fester Stoffe, Nöthnitzer Str. 40, 01187 Dresden, Germany

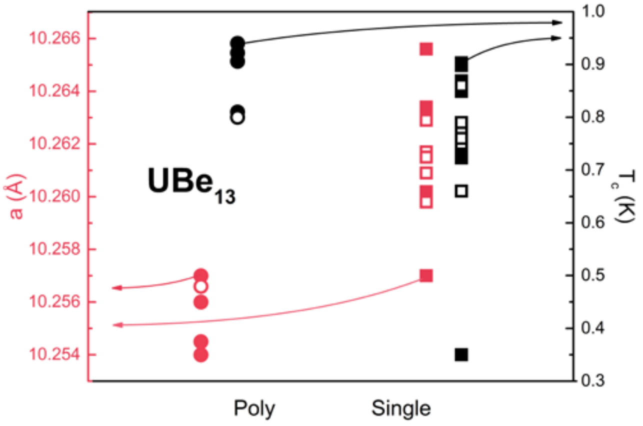

Figure S 1. Lattice parameter a and T_c for UBe_{13} polycrystalline samples (round symbols) and single crystals (squares) from literature reports (full symbols) and this work (empty symbols. Literature data taken from refs. [1-13]

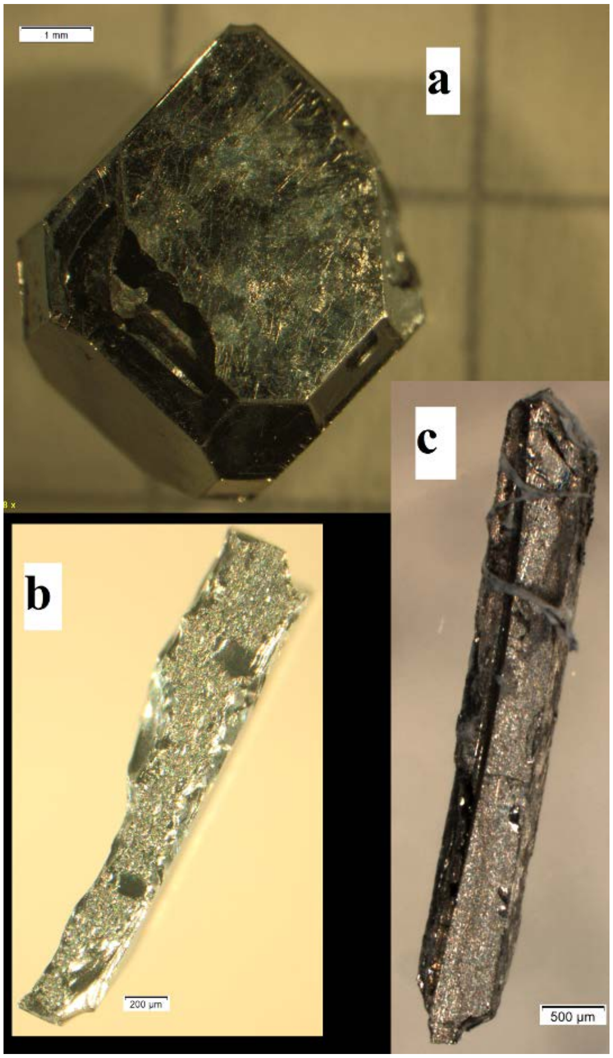

Figure S 2. UBe_{13} single crystal in as-grown state (a), the medium-sized piece (b) and the large piece (c) cut from it.

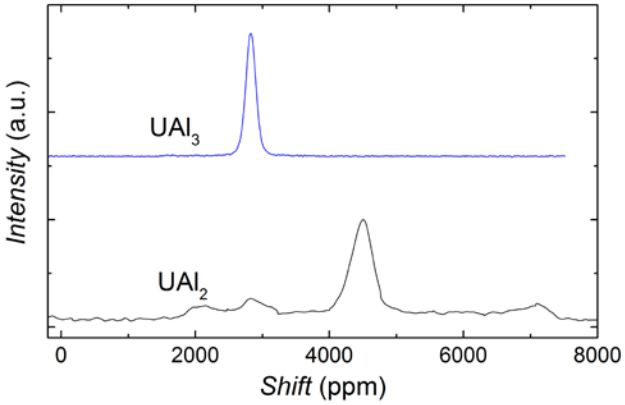

Figure S 3. ^{27}Al-NMR (MAS) spectra of powdered polycrystalline samples of UAl_3 and UAl_2 (containing a small signal from UAl_3 impurities).

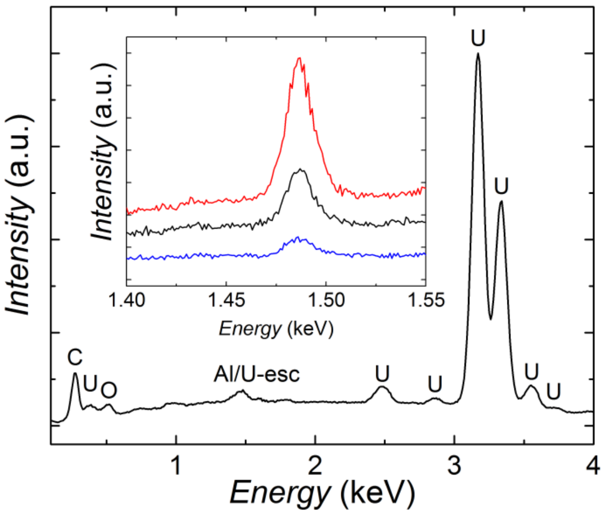

Figure S 4. EDX spectrum recorded on a cleaved surface of a $UBe_{13-x}Al_x$ single crystal and WDX (inset) spectra recorded on three different single crystals of $UBe_{13-x}Al_x$.

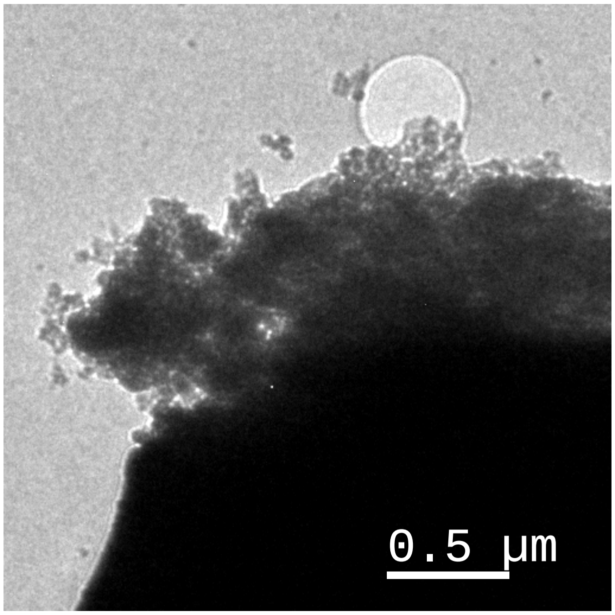

Figure S 5. TEM image showing the disintegration of a UBe_{13} single crystal on the nanometer scale, observed on several crystallites after 76 d of annealing at 900 °C.

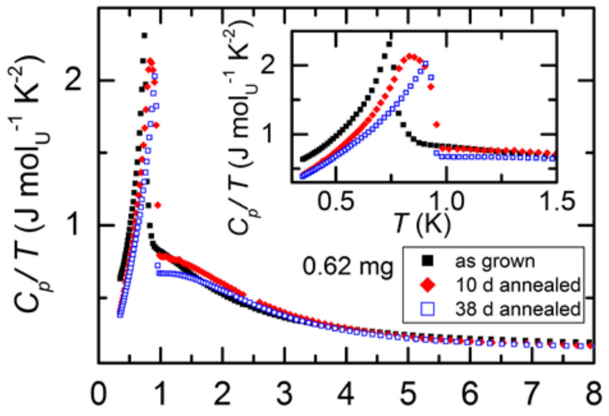

Figure S 6. C_p/T vs. T for the small crystal for several stages of annealing.

Table S 1. Polycrystalline samples of the $UBe_{13-x}Al_x$ series with measured lattice parameters *a*.

at% Al	x in $UBe_{13-x}Al_x$	a (Å), as cast	a (Å), annealed (27d, 900 °C)
0	0	10.2566(1)	
0.20	0.03	10.2600(2)	10.2566(2)
0.51	0.07	10.2606(2)	10.2573(2)
0.78	0.11	10.2664(1)	10.2571(2)
2.1	0.29	10.2736(1)	10.2584(1)
3.59	0.50	10.2762(2)	10.2633(1)
4.2	0.60	10.2775(1)	10.2633(1)

Table S 2. Investigated samples of UBe_{13} with lattice parameter *a* and T_c where measured.

Composition	State	*a* (Å)	T_c (K)
UBe_{13}, polycryst.	as-cast	10.2566(1)	0.80
U_5Be_{95}, polycryst.	as-cast	10.2569(2)	0.82
$U_{10}Be_{90}$, polycryst.	as-cast	10.2564(2)	0.85
$UBe_{13-x}Al_x$, single crystal	as-grown	10.2617(2)	0.75
$UBe_{13-x}Al_x$, single crystal	as-grown	10.2615(3)	
$UBe_{13-x}Al_x$, single crystal	as-grown	10.2609(2)	0.77
$UBe_{13-x}Al_x$, single crystal	as-grown	10.2630(2)	0.78
$UBe_{13-x}Al_x$, single crystal	as-grown	10.2629(1)	0.66

Table S 3. The peak maxima in C_p/T_{max} at the superconducting transition, the electronic specific heat coefficient γ and the critical temperature T_c for single crystals and polycrystalline samples, collected from literature reports and our own data.

T_c (K)	C_p/T_{max} (J mol_U^{-1} K^{-2})	γ (J mol_U^{-1} K^{-2})	Material	Source
0.83	2.7	0.7	Polycrystalline	[1]
0.83	2.4	0.8	Polycrystalline	[2]
0.88	2.5	0.8	Polycrystalline	[3]
0.85	2.3	0.8	Polycrystalline	[4]
0.75	2.2	0.7	Polycrystalline	[5]
0.88	2.5	0.8	Polycrystalline	[6]
0.7	2.1	1.0	Polycrystalline	[7]
0.8	2.2	0.7	Polycrystalline	[8]
0.78	2.2	0.7	Polycrystalline	[9]
0.87	2.4	0.8	Polycrystalline	[10]
0.83	2.5	0.8	Polycrystalline	[11]
0.83	2.1	0.75	Polycrystalline	[12]
0.9	2.8	0.9	Single crystal	[13]
0.72	3.3	1.1	Single crystal	[14]
0.87	2.8	0.8	Single crystal	[14]
0.73	1.9	0.8	Single crystal	[15]
0.72	2.8	1.1	Single crystal	[16]
0.7	3.6	1.1	Single crystal	[12]
0.68	3.5	1.2	Single crystal	[17]
0.78	2.8	0.9	Single crystal	[18]
0.8	3.4	1.0	Single crystal	[19]
0.74	2.01	0.76	Polycrystalline	Own data
0.66	1.82	0.8	Polycrystalline	Own data
0.78	2.26	0.86	Polycrystalline	Own data
0.78	1.97	0.79	Polycrystalline	Own data
0.78	2.21	0.9	Polycrystalline	Own data
0.65	1.79	0.72	Polycrystalline	Own data
0.74	2.31	0.85	Single crystal	Own data
0.84	2.13	0.80	Single crystal	Own data
0.91	2.24	0.72	Single crystal	Own data
0.62	3.49	1.09	Single crystal	Own data
0.70	2.28	0.97	Single crystal	Own data
0.78	2.93	0.97	Single crystal	Own data
0.93	2.04	0.6	Single crystal	Own data
0.92	1.73	0.68	Single crystal	Own data
0.67	3.81	1.22	Single crystal	Own data
0.67	2.93	1.02	Single crystal	Own data
0.75	2.65	0.98	Single crystal	Own data
0.79	2.60	0.97	Single crystal	Own data
0.80	2.59	0.94	Single crystal	Own data

References for Supporting Information

[1] J. P. Brison et al., "Normal and superconducting properties of UBe13," *J. Phys. France*, vol. 50, pp. 2795-2810, 1989. [Online]. https://doi.org/10.1051/jphys:0198900500180279500

[2] B. Ellman, T. F. Rosenbaum, J. S. Kim, and G. R. Stewart, "Thermodynamic features in the H-T plane of superconducting UBe_13," *Phys. Rev. B*, vol. 44, no. 21, pp. 12074-12076, Dec 1991. [Online]. https://link.aps.org/doi/10.1103/PhysRevB.44.12074

[3] Z. Fisk, J. L. Smith, H. R. Ott, and B. Batlogg, "Heavy fermion behavior in uranium compounds," *Journal of Magnetism and Magnetic Materials*, vol. 52, pp. 79-84, 1985. [Online]. https://doi.org/10.1016/0304-8853(85)90229-X

[4] M. J. Graf, N. A. Fortune, J. S. Brooks, J. L. Smith, and Z. Fisk, "Specific heat of pure and thoriated UBe13 at low temperatures in high magnetic fields," *Phys. Rev. B*, vol. 40, no. 13, pp. 9358-9361, Nov 1989. [Online]. https://link.aps.org/doi/10.1103/PhysRevB.40.9358

[5] H. M. Mayer et al., "Normal-state and superconducting properties of the heavy-fermion compound UBe13 in magnetic fields," *Phys. Rev. B*, vol. 33, no. 5, pp. 3168-3171, Mar 1986. [Online]. https://link.aps.org/doi/10.1103/PhysRevB.33.3168

[6] H. R. Ott et al., "p-Wave superconductivity in UBe13," *Phys. Rev. Lett.*, vol. 52, pp. 1915-1918, 1984. [Online]. https://doi.org/10.1103/PhysRevLett.52.1915

[7] H. A. Radovan, R. J. Zieve, J. S. Kim, and G. R. Stewart, "Implications of Tc-Variation in UBe13 for a Possible Fulde--Ferrell--Larkin--Ovchinnikov Phase," *Journal of Superconductivity*, vol. 16, pp. 957-960, Dec 2003. [Online]. https://doi.org/10.1023/A:1026231121310

[8] U. Rauchschwalbe, C. D. Bredi, F. Steglich, K. Maki, and P. Fulde, "Phase Diagram of the Superconducting States of U1-xThxBe13," *Europhysics Letters*, vol. 3, p. 757, 1987. [Online]. http://stacks.iop.org/0295-5075/3/i=6/a=017

[9] A. Ravex, J. Flouquet, J. L. Tholence, D. Jaccard, and A. Meyer, "Thermal conductivity and specific heat measurements on UBe13," *Journal of Magnetism and Magnetic Materials*, vol. 63-64, pp. 400-402, 1987. [Online]. http://www.sciencedirect.com/science/article/pii/0304885387906214

[10] E. A. Schuberth, K. Kloss, J. Schupp, and K. Andres, "Specific heat and magnetic susceptibility of UPt3 and UBe13 at mK-temperatures," *Z. Phys. B Con. Mat.*, vol. 97, pp. 55-58, Mar 1995. [Online]. https://doi.org/10.1007/BF01317587

[11] Yusei Shimizu, Alexandre Pourret, Georg Knebel, Alexandra Palacio-Morales, and Dai Aoki, "Non-Fermi-liquid nature and exotic thermoelectric power in the heavy-fermion superconductor UBe13," *Phys. Rev. B*, vol. 92, no. 24, p. 241101, Dec 2015. [Online]. https://link.aps.org/doi/10.1103/PhysRevB.92.241101

[12] A. P. Ramirez, C. M. Varma, Z. Fisk, and J. L. Smith, "Fermi-liquid renormalization in the superconducting state of UBe13," *Philosophical Magazine B*, vol. 79, pp. 111-117, 1999. [Online]. https://doi.org/10.1080/13642819908206785

[13] F. Kromer et al., "UBe13: Another phase transition below Tc?," *Chinese Journal of Physics*, vol.

36, p. 157, Apr. 1998.

[14] C. Langhammer et al., "Evidence for the existence of two variants of UBe13," *J. Magn. Magn. Mater.*, vol. 177-181, pp. 443-444, 1998. [Online]. https://doi.org/10.1016/S0304-8853(97)00690-2

[15] C. F. Miclea et al., "Unconventional superconductivity in UBe13 probed by uniaxial stress," *Physica B: Condensed Matter*, vol. 312-313, pp. 97-99, 2002, The International Conference on Strongly Correlated Electron Systems. [Online]. https://doi.org/10.1016/S0921-4526(01)01071-7

[16] H. R. Ott, H. Rudigier, Z. Fisk, and J. L. Smith, "UBe13: An unconventional actinide superconductor," *Phys. Rev. Lett.*, vol. 50, pp. 1595-1598, 1983. [Online]. https://link.aps.org/doi/10.1103/PhysRevLett.50.1595

[17] P. H. P. Reinders et al., "Specific-Heat Measurements on UBe13 under Uniaxial Pressure," *Europhysics Letters*, vol. 24, pp. 619-624, 1994. [Online]. https://doi.org/10.1209/0295-5075/25/8/010

[18] Yusei Shimizu et al., "Maki Parameter and Upper Critical Field of the Heavy-Fermion Superconductor UBe13," *Journal of the Physical Society of Japan*, vol. 80, p. 093701, 2011. [Online]. https://doi.org/10.1143/JPSJ.80.093701

[19] Yusei Shimizu et al., "Heat capacity measurements on UBe13 in rotated magnetic fields: Anisotropic response in the normal state and absence of nodal quasiparticles," *Journal of Magnetism and Magnetic Materials*, vol. 400, pp. 52-55, 2016, Proceedings of the 20th International Conference on Magnetism (Barcelona) 5-10 July 2015. [Online]. http://www.sciencedirect.com/science/article/pii/S0304885315304273
